# The paradoxical effects of professional stereotypes on the quality of care by interprofessional teams: The contingent effects of team faultlines, team stereotypes, and championship behaviors

**DOI:** 10.3389/fpsyg.2023.1135071

**Published:** 2023-03-14

**Authors:** Galia Sheffer Hilel, Anat Drach-Zahavy, Ronit Endevelt

**Affiliations:** ^1^Nutrition Sciences Department, Faculty of Sciences at Tel-Hai College, Kiryat Shmona, Israel; ^2^Nursing Department, Faculty of Social Welfare and Health Sciences at Haifa University, Haifa, Israel; ^3^School of Public Health, Faculty of Social Welfare and Health Sciences at Haifa University, Haifa, Israel

**Keywords:** Interprofessional teams, stereotypes, leadership, championship behaviors, faultlines

## Abstract

**Background:**

Despite calls for interprofessional teamwork to ensure quality care in healthcare settings, interprofessional teams do not always perform effectively. There is evidence that professional stereotypes inhibit effective interprofessional teamwork, but they haven’t been explored as a phenomenon that impacts team’s performance and quality of care.

**Objectives:**

To focus on professional stereotypes emerging in interprofessional teams and examine the contingency effects of interprofessional team’s faultlines, professional stereotypes, and leader’s championship behaviors on team’s quality of care.

**Methods:**

A cross-sectional nested sample of 59 interprofessional teams and 284 professionals, working in geriatric long-term-care facilities in Israel. Additionally, five to seven of the residents of each facility were randomly sampled to obtain the outcome variable. Data collection employed a multisource (interprofessional team members), multimethod (validated questionnaires and data from residents’ health records) strategy.

**Results:**

The results indicated that faultlines are not directly harmful to team’s quality of care; instead, they are likely to impact quality of care only when team stereotypes emerge. Furthermore, whereas teams typified by high professional stereotypes require person-oriented championship leadership, for teams typified by low team stereotypes, championship leadership harms the quality of care they provide.

**Conclusion:**

These findings have implications for handling interprofessional teams. Practically, leaders must be well-educated to better analyze team members’ needs and maintain the appropriate leadership style.

## Introduction

As healthcare settings pursue new reforms to ensure patients’ safe, high-quality care, the need to collaborate *via* interprofessional teamwork grows substantially (Freund and Drach-Zahavy, 2007; Winstein et al., 2016). Scholars view the interdisciplinary team as a “proxy for cognitive heterogeneity, representing innovativeness, problem-solving abilities, creativity, diversity of information sources and perspectives, openness to change, and willingness to challenge and be challenged” (Finkelstein et al., 2009, p. 125). Concomitantly, the World Health Organization (2010) called for adopting “a different paradigm in the management of health personnel through evidence-based policies and practices that promote collaborative interprofessional teamwork.” Advocates of employing healthcare interprofessional teams argue that, in combination, they contain a more comprehensive information base, equipping the team to develop innovative solutions to complex patient- and service-related challenges (Dias and Escoval, 2013; Mitchell and Boyle, 2015).

Despite these repeated calls for administrators, policymakers, and scholars to advance interprofessional care, research findings so far have been inconclusive, suggesting that interprofessional teams do not necessarily fulfill their potential to perform effectively, as they may experience friction, hostility, and poor performance (Mitchell and Boyle, 2015, 2019; Homan et al., 2020). Apparently, the obvious professional diversity of the interprofessional team members, coupled with additional potential diversity for other attributes (e.g., race, age, educational background), can sometimes hamper team performance and quality of care (Sarma et al., 2012).

A key cause of interprofessional teamwork failure is the emergence of professional stereotypes–cognitive structures that provide knowledge, beliefs, and expectations about individuals, based on their belongingness to a profession (Quadflieg and Macrae, 2011). Stereotypes may be positive (e.g., all social workers are compassionate) or negative (e.g., physicians are poor team leaders), but in most cases harm the effectiveness of the interprofessional team, particularly in cases where there is a need for in-depth information elaboration on novel tasks (Meyer et al., 2022). In most cases, stereotypes trigger negative intrateam interactions, conflict, distrust, disliking, and limited communication among interprofessional team members, thereby perhaps challenging the foundation for creating the interprofessional team in the first place, and harming team effectiveness (van Dijk et al., 2017; Conroy, 2019).

**Figure 1:**
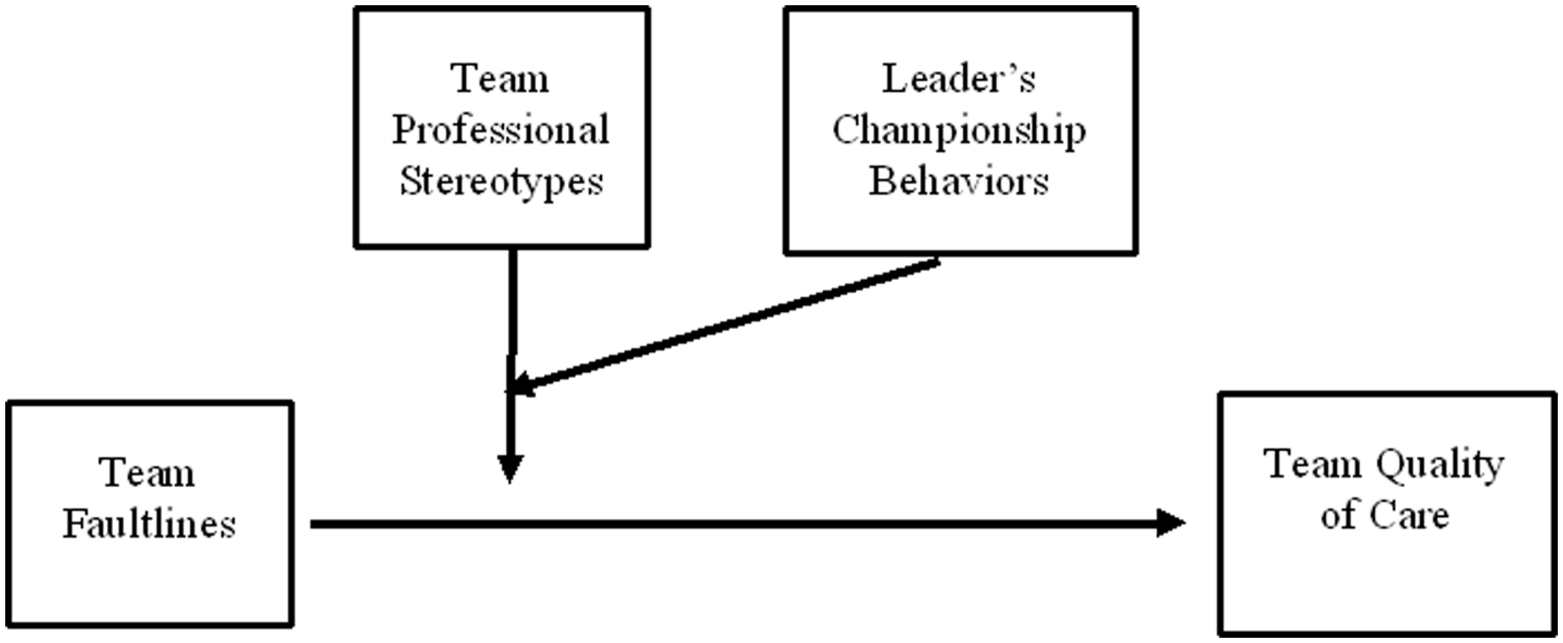
The study model.

This study focuses on professional stereotypes emerging in interprofessional teams and aims to explore the circumstances where stereotypes impede teamwork and the means to buffer those harmful effects. Embedded within the categorization-elaboration model (CEM; van Knippenberg et al., 2004) combined with the leadership diversity model LeaD (Homan et al., 2020), we suggest that team diversity (in terms of team faultlines), team’s professional stereotypes, and the leader’s championship behaviors interact in their impact on the team’s quality of care (Figure 1).

CEM proposes that interprofessional teamwork might be characterized by two alternative potentially complementary pathways (van Knippenberg et al., 2004). According to the first—*the elaboration of information pathway,* in the absence of professional stereotypes, interprofessional team diversity can increase the effectiveness of team quality of care. LeaD similarly suggests that under these circumstances, person-related leadership (championship behaviors) may be redundant and can even harm the team’s quality of care (Homan et al., 2020).

Alternatively, in line with the second pathway—*the team categorization pathway*—when professional stereotypes emerge within a team, the team leader is required to intervene actively to attenuate any interpersonal conflicts and/or inadequate communication among the interprofessional team members. Here is where the LeaD model (Homan et al., 2020) can contribute by suggesting that leadership style is contingent on the interprofessional team’s needs and thus should differ substantially between interdisciplinary teams that face intergroup bias and those that engage with information elaboration. Accordingly, person-related leadership may be required to attenuate the deteriorating effects of team professional stereotypes on team effectiveness. Thus, the leader’s championship behaviors, as a form of person-related leadership, encompassing expressions of enthusiasm for team success and perseverance under adversity, in tandem with individualized attention to each member’s contribution and involving the appropriate people, might lessen the negative impact of emerging stereotypes. We test this model (Figure 1) with interdisciplinary team members working in 59 geriatric long-term-care facilities (LTCFs) that implement reforms to improve residents’ quality of care.

## Background and hypotheses testing

### Professional stereotypes

Practitioners on an interprofessional team are acculturated into professional groups; thus, they may often develop professional stereotypes, thereby creating barriers to collaboration (Hall, 2005). Embedded within the social identity framework (van Knippenberg and Schippers, 2007; van Dick et al., 2008) and its extension, professional identity theory (Schein, 1978), scholars argue that the professionally diverse composition of the interprofessional team reinforces the salience of professional identity by increasing the cognitive accessibility of profession as a social category (Mitchell and Boyle, 2015). Consequently, categorization processes among professional groups might create stereotypes about other professional groups (Hean et al., 2006; Mitchell et al., 2011).

Professional stereotypes are overgeneralized representations of a group of people based on their profession (Conroy, 2019). Through professional stereotyping, one infers characteristics from a single professional, such as a nurse or a social worker, assuming that all members of the profession also possess these traits. No matter how positive or negative stereotypes are, they most frequently lead to prejudice against the professional group (Conroy, 2019). In healthcare, professional stereotypes are reinforced through institutionalized mechanisms (e.g., the medical hierarchy; Thylefors, 2012), which leads to distrust and conflict among professions, thus hindering communication between them (McNeil et al., 2013). Early research on professional stereotypes of healthcare professionals has been mainly descriptive. Studies depicted hetero-stereotypes (stereotypes of other professions against one’s profession) or auto-stereotypes (stereotypes of one’s own profession against one’s profession) among practitioners (Barnes et al., 2000; Kämmer and Ewers, 2022) and students (Foster and Macleod Clark, 2015). Previous studies also evaluated the impact of interprofessional education (IPE) interventions of various types and durations on the reduction of professional stereotypes, with mixed results (Barnes et al., 2000; Michalec et al., 2013). More recently, Conroy (2019) argued that professional stereotypes might represent a barrier to interprofessional team outcomes, as stereotyping increases the risk of breakdowns in communication and coordination. This is unfortunate because the main rationale for using interprofessional teams is to increase the communication and coordination among professionals who care for patients (Zwarenstein et al., 2009; Gittell et al., 2013). A theoretical model, delineating the boundary conditions that determine whether and how interpersonal stereotypes create long-term consequences in team functioning has recently been introduced (van Dijk et al., 2017). Still, this question has gained limited empirical attention so far.

Further, professional stereotypes have been typically addressed as individually manifested states, cognitions, and acts that may have a direct bearing on interpersonal relationships. However, part of the professional-stereotypes phenomenon may be further understood by investigating how it is embedded in different contexts, such as the interprofessional team. The input-process-output–input (IPOI) framework that dominates current team research (Ilgen et al., 2005; Mathieu et al., 2014) provides solid theoretical ground for considering professional stereotypes as a relatively shared property of the team. Accordingly, team members are exposed to similar inputs in their work environment, such as organizational structures that separate professional groups, or how leaders, colleagues, and patients react to different types of professionals. The team members then share their interpretations of the inputs with each other, creating emergent states or team processes based on the shared interpretations. This leads to similar reactions and behaviors among team members (Weick, 1993; Lindell and Brandt, 2000; Mathieu et al., 2014). Here, we examine professional stereotypes as overgeneralized representations of a group of people based on their profession and their moderating effect on team effectiveness.

### The joint effects of team diversity and team’s professional stereotypes

Obviously, the interprofessional team is by definition diverse in terms of professions. Yet, faultline theory (Lau and Murnighan, 1998; Chrobot-Mason et al., 2009) argues that in considering the impact of team diversity, other dimensions of diversity (e.g., tenure, gender) should also be considered. Apparently, the impact of diversity is stronger when members differ from each other in the same way on more than one attribute Van Dijk (e.g., when dietitians in the interprofessional team are also women and younger than physicians, who are also men, and older; Meyer et al., 2014; van Dijk et al., 2017). In line with van Knippenberg et al.’s (2004) CEM model, team faultlines are not fundamentally “good” or “bad”; instead, they can enact two distinct, yet not mutually exclusive, pathways: team categorization processes and information elaboration.

As for the former, as an extension of the classic self-categorization model (Tajfel et al., 1986), CEM (van Knippenberg et al., 2004) contends that team diversity may be linked to harmful outcomes only when (a) team diversity attributes serve as a basis for categorization processes (i.e., the perception of subgroups) and (b) categorization further creates intergroup bias, namely favoring one’s own subgroup while out-group members are subject to projected biases and prejudices, and tend to be excluded from formal and informal interaction. These joint circumstances may create professional stereotypes, hindering the care provided to patients.

Alternatively, if information elaboration is prominent, intergroup bias and stereotypes are less likely to surface, thus improving team effectiveness, and the quality of care provided to patients. Information elaboration refers to “the degree to which information, ideas, or cognitive processes are shared, and are being shared, among the group members” (Hinsz et al., 1997, p. 43) and involves “feeding back the results of […] individual-level processing into the group, and discussion and integration of their implications” (Homan et al., 2007, p. 1,189). Yet, as our model suggests, this link may be moderated by team’s championship behaviors.

### Championship behaviors, team faultlines, and team’s professional stereotypes

The notion that the impact of team faultlines could be mitigated by the leader’s behavior is not new and has attracted ample research (Meyer et al., 2015; Homan et al., 2020). In the context of healthcare, champions of innovation play increasingly important roles in leading the quality of care of interprofessional teams (Howell et al., 2005; Byers, 2017). They are exceptional frontline practitioners who are formally or informally nominated to lead their teams and who are passionate and dedicated to working on improving the quality of care in the teams (Schon, 1963; Howell and Higgins, 1990). Champions are characterized by three main person-focused behaviors: expressing optimism and confidence about the team’s success, building networks by assembling the right interprofessional-team members, and persisting despite the difficulties (Howell et al., 2005; Luz et al., 2019b). These behaviors are aimed at facilitating the social interactions among team members and their motivational attitudes such that effective teamwork is enabled (Homan et al., 2020).

Yet, preliminary empirical evidence may suggest that championship behaviors do not always benefit team success and that sometimes the champion may even disrupt team effectiveness (Markham et al., 1991; Pinto and Patanakul, 2015). For example, in a recent study of 94 medical wards, Luz et al. (2019a) concluded that championship behaviors facilitated the novelty of team projects only when team members’ engagement and enthusiasm were required. In contrast, championship behaviors did not improve and even hampered novelty when projects required tighter supervision and leader’s monitoring (Luz et al., 2019a). Under these circumstances, championship behaviors might have been redundant and even harmful (Walter et al., 2011). A recent research review, summarizing findings on a broad variety of leadership styles (e.g., inclusive leadership, transformational leadership, inspirational leadership; Homan et al., 2020), reached similar conclusions, serving as the impetus for the development of the LeaD model (Homan et al., 2020). Briefly, the model proposes that leadership style is contingent on the interdisciplinary team’s needs and thus should differ substantially between interprofessional teams that face intergroup bias and those that engage with information elaboration. Whereas the former teams require the leader’s person-focused behaviors that establish the social interactions and motivations, necessary to enable effective teamwork, the latter teams demand the leader’s task-oriented behaviors that facilitate the understanding of task requirements, procedures, and the acquisition of task-relevant information (Homan et al., 2020). Similarly, the substitute-for-leadership theory argues that certain individual, task, and organizational variables act as “substitutes for leadership,” thereby negating the leader’s ability to influence team members’ effectiveness (Kerr and Jermier, 1978).

Accordingly, we propose that interprofessional teams facing professional stereotypes require people-oriented leadership behaviors (e.g., championship behaviors), aimed at proactively preventing or retroactively suppressing stereotypes. Apparently, by expressing confidence in the team’s success, the champion conveys that the team’s achievements are a mutual goal that is attainable regardless of professional belongingness; thus, input from all members, especially those who may not usually participate in discussions, is welcomed. Moreover, by building networks and assigning the right people to the right tasks without prejudice, the champion expresses the value in diverse, even conflicting, opinions from different professions and signals that all are perceived as equally important members of the team. Champions who incorporate higher levels of these behaviors are likely to engender an atmosphere of mutual respect across the different professions, in which the specialized expertise held by each professional is perceived as valuable to the team’s shared task (Mitchell et al., 2015; Mitchell and Boyle, 2019).

Conversely, when the interprofessional teamwork is not accompanied with professional stereotypes, and thus is likely to engage in information elaboration, person-focused behaviors are redundant or even harmful. Under these circumstances, the team requires more task-structuring behaviors such as establishing and monitoring task deadlines and goals for the different stages of a project, as well as hands-on provision of task information and training to achieve those deadlines and goals (Luz et al., 2019a; Homan et al., 2020).

To conclude, we propose that the three-way interaction of team’s faultlines, professional stereotypes, and championship behaviors will have a significant relationship to team’s quality of care. The rationale behind this suggestion is that team faultlines do not necessarily deteriorate team quality, but only when team stereotypes emerge (van Knippenberg et al., 2004). Likewise, team stereotypes do not necessarily harm the quality of care provided by the team. For teams typified by high team professional stereotypes—championship behaviors, exhibited by the team leader, may be helpful in easing the harmful impacts of the stereotypes, whereas in teams with low team stereotypes, the championship behaviors exhibited by the team leader may be redundant and even harmful (Homan et al., 2020).

Accordingly, we propose the following:

The three-way interaction of championship behaviors, faultlines, and professional stereotypes will have a significant relationship to team’s quality of care, such that

(a) when the team’s professional stereotypes are high, team quality of care will be associated with championship behaviors regardless of the level of faultlines;
(b) when the team’s professional stereotypes are low, team quality of care is contingent upon championship behaviors such that.
(b1) when championship behaviors are low, the stronger the team faultlines the higher the team’s quality of care;
(b2) when championship behaviors are high, the stronger the team faultlines the lower the team’s quality of care.

## Methods

### Setting

The Ministry of Health’s nutrition division launched a program aimed at improving the quality of care of LTCF residents in Israel. The program trained dietitians to lead residents’ oral health reform by conducting a Nutrition-Focused Physical Examination (NFPE) with all residents of LTCFs, developing an interprofessional care plan, and engaging the interprofessional team members at the facility to improve residents’ quality of care. The main assumptions underlying the program were that (a) preserving and improving residents’ quality of life and nutrition status requires interdisciplinary teamwork that addresses oral health and swallowing problems and (b) the dietitian as a champion of innovation should lead the program in the various LTCFs (Weening-Verbree et al., 2013). Accordingly, the dietitian, who is the champion of innovation, would conduct the NFPE and, based on the results, prepare a nutritional intervention that brings together all interprofessional team members. The physician would treat abnormal cases and adjust the drug treatment if xerostomia or a taste/smell change was found. The nurse, as responsible to the nonprofessional care workers, would be responsible for residents’ oral hygiene and would instruct the staff on food-serving modes. The speech therapist would diagnose and determine the texture of food and fluids. The occupational therapist would provide tools to improve eating abilities. The social worker would seek funding options for dental treatments. The physiotherapist would address sitting and head positions while eating. Hence, there is a connection between the dietitian’s examination results for oral health and the interprofessional team’s approach. Protocols were developed for integrating the program into routine work, and infrastructure was formed to document the information in residents’ electronic health records (EHRs).

### Design

The study employed a cross-sectional nested design, where 284 professionals were nested within 59 LTCFs.

### Sample and study procedure

All LTCFs in rural and urban areas throughout Israel were invited to participate in the study. The inclusion criteria included an LTCF where the dietitian was exposed to the new program. Exclusion criterion was institutional tenure of at least 1 year for each team member. Eleven wards declined. Thus, the final sample included 59 LTCFs (participation rate: 84%). Of these, most were medium-size LTCFs (*n* = 25; 44.8%), followed by small institutions (*n* = 17; 28.8%) and large institutions (*n* = 17; 28.1%). Most LTCFs (79.6%) were for-profit and the rest were nonprofit (20.4%).

In total, 284 interdisciplinary professionals working in 59 LTCFs completed the questionnaires (response rate = 70%), including 57 physicians, 59 nurses, 36 physiotherapists, 33 social workers, 28 occupational therapists, 12 speech therapists, and 59 dietitians (between 4 and 7 different professionals in a LCTF). Their ages ranged from 24 to 78 years (*M* = 44.65, *SD* = 13.65). The sample included 76.4% females; their institutional tenure ranged from 1 to 25 years (*M* = 6.21, *SD* = 5.54); and 62.9% held a bachelor’s degree, 17.3% a master’s degree, and the rest held a doctoral degree. Estimation of the required sample size was made using alpha = 0.05 and group sizes of 4–7 participants; It indicated the need for a sample size of *n* = 230 for level 1 and *n* = 55 for level 2 to ensure a power of at least 0.80 and effect size of at least 0.4 for all our hypotheses.

LTCF managers received a letter explaining the study and its objectives. After obtaining their consent, the researcher met with the interdisciplinary teams at their institutions to complete the questionnaires. Two weeks later, residents’ information was collected from EHRs.

### Data collection

Data were collected during 2019. To decrease bias, we employed a multisource (interprofessional team members and residents), multimethod (validated questionnaires, EHRs) strategy for data collection (Podsakoff et al., 2003).

*Team’s quality of care,* the dependent variable, was assessed using data gathered from EHRs of the 5–7 randomly selected residents for whom the team cared (*n* = 292 residents). Only new residents (hospitalized for 1 to 8 months) were included, as the protocol requires each to undergo dietitian assessment. Terminal patients or patients receiving enteral-tube feeding were excluded. Data were gathered *via* a checklist, developed, and validated specifically for the present study. The checklist was designed to assess the extent to which the dietitian, as project champion, succeeded in engaging the interprofessional team in the project. Observing residents’ information in the EHRs, the researcher assessed on a 4-point Likert-type scale (0 = *not performed at all*; 1 = *low partial performance*; 2 = *high partial performance*; 3 = *fully performed*) whether there was a record of the dietitian’s recommendations to the interdisciplinary team members in accordance with the findings, whether the interprofessional team members performed the recommendation, and whether there was a record of monitoring the performance of the recommendations by the interdisciplinary team members and evaluation of their work. Quality of care was calculated as the mean score across residents in a particular LTCF.

To validate a team’s quality of care, we conducted a pilot study with 20 experts of NFPE, serving as managers in LTCFs, as supervisors at the Ministry of Health, or in academia. All were women; their ages ranged from 32 to 65 years (mean [*M*] = 47.2, standard deviation [*SD*] = 11), and their seniority ranged from 5 to 40 years (*M* = 21.75, *SD* = 10). To examine the face validity of the success score, capturing the extent to which the scale’s questions reflected our intended measures, we asked the experts to assess their clarity: “Are all the questionnaire items clear?;” “Should items be added or removed to cover the subject?” These served as criteria for modifying items. Consequently, we clarified that the items refer to health situations in which the patient would need care from other members of the interprofessional team.

To establish content validity, we asked the 20 experts to rate, on a 5-point Likert-type scale, the relevance of each indicator to quality-of-care implementation (1 = *not relevant*; 5 = *very relevant*). We calculated the content validity index (CVI), defined as the proportion of items rated as quite/very relevant by each expert. The CVI score was 0.90, indicating good validity (Polit et al., 2007). Then, to test interrater reliability, three dietitians separately evaluated the success measure with a sample of 20 residents. There was full agreement between their evaluations. Finally, to test the criterion validity, we calculated the association between our new measure and a well-established questionnaire of team effectiveness by the ward manager and found a significant positive correlation (*r* = 0.0.368, *p* < 0.001).

*Team diversity* was assessed in line with faultline theory using the average silhouette width (ASW) method (Meyer and Glenz, 2013). To determine the strength of the team faultlines, we calculated the ASW across the three most commonly discussed attributes in the faultlines literature: gender, academic degree, and team tenure (e.g., Carton and Cummings, 2012). We did not include team members’ age because of its high correlation with team tenure (*r* = 0.42, *p* < 0.01). The literature (e.g., Meyer and Glenz, 2013; Meyer et al., 2014) indicates that the alignment of attributes that are highly correlated should be avoided because the redundant information will bias the estimate. ASW was calculated using R with the ASW cluster package for faultline calculation (Meyer and Glenz, 2013). Faultline strength ranges from 0 to 1, where values closer to 1 represents maximum alignment of multiple attributes, resulting in maximum separation of a group into homogeneous subgroups.

*Team’s professional stereotypes* were measured using the Student Stereotype Rating Questionnaire (SSRQ; Hean et al., 2006). The questionnaire addresses nine characteristics: academic ability, professional competence, interpersonal skills (i.e., warmth, sympathy, communication), leadership abilities, ability to work independently, ability to be a team player, ability to make decisions, practical skills, and confidence. Members of the interprofessional team were asked to assess on a 7-point Likert-type scale (1 = *very low*; 7 = *very high*) the extent to which they believed each profession (nurses, physicians, dietitians, social workers, occupational therapists, and speech therapists) is characterized by the attribute. To obtain the individual professional stereotypes score, we first calculated the mean score that each individual provided for the seven professions. Next, in line with the definition of professional stereotypes as “cognitive structures that provide knowledge, beliefs, and expectations about individuals based on their belongingness to a profession” (Quadflieg & Macrae, 2011, p. 216–217), we calculated the individual member’s level of stereotypes as the *SD* of the mean ratings across professions. A high *SD* indicates high stereotypes, as the individual assigned attributes to professionals according to their profession. In comparison, a low *SD* indicates low stereotypes, and that the profession does not serve as a criterion for assessing attributes. To calculate team-level professional stereotypes, we averaged individual professional stereotypes across team members. To assess professional stereotypes, we averaged the interprofessional team members’ evaluations, ensuring the appropriateness of our aggregations with ICC scores (James, 1982). The findings indicated that ICC(1) = 0.12 and ICC(2) = 0.56, showing satisfactory results.

*Championship behavior* was assessed with Howell et al.’s (2005) 15-item questionnaire, comprising three subscales. Interprofessional team members were asked to rate the dietitian’s championship behaviors on a 7-point frequency scale (1 = *never*; 7 = *always*). Six items measure expressing enthusiasm for and confidence in the innovation’s success: for example, “[the dietitian] expresses confidence in what the innovation can do” (ɑ = 0.961); six items measure persistence under adversity: for example, “[the dietitian] persists in the face of adversity” (ɑ = 0.961); and three items measure network-building by involving the right people: for example, “[the dietitian] gets key decision-makers involved” (ɑ = 0.941). Total alpha reliability across the three subscales was 0.97. To assess dietitian’s championship behavior, we averaged the interprofessional team members’ evaluations, ensuring the appropriateness of our aggregations with ICC scores (James, 1982). The findings indicated that ICC (1) = 0.34 and ICC (2) = 0.76, showing satisfactory results.

#### Control variables

We also collected interprofessional team members’ sociodemographic characteristics: academic degree (bachelor’s/master’s/doctorate), gender, and team’s tenure, and organizational characteristics: institution type (nonprofit/for-profit) and size (number of beds: small [>36], medium [36–180], and large [<180]).

### Data analysis

Data analysis was conducted in three steps using SPSS, version 23.0 (IBM Corp., Armonk, NY, United States). First, descriptive analyses were presented, including means and SDs for continuous variables, and percentages for nominal variables. Next, we conducted univariate analyses: Pearson’s correlations for continuous variables and t-tests and ANOVA for ordinal variables to provide preliminary support for our hypotheses. Third, prior to the hypotheses testing, we employed the Kolmogorov–Smirnov test and Monte Carlo calculations. These tests were non-significant, supporting the adequacy of Mixed linear model analyzed to analyze our data. We employed a mixed linear model analysis because of the nested sample: residents were nested in naturally occurring hierarchies (Singer, 1998). We followed the procedure recommended (Baron and Kenny, 1986) for testing moderating models. Accordingly, the control and the interdependent variables were entered into the first step, all two-way interactions were entered into the second step, and three-way interactions into the third step.

### Ethical considerations

Participants signed informed consent forms, and data confidentiality was ensured. Because of the need to link the dietitians’, interdisciplinary team members’, and residents’ data, the study was not anonymous. Each interdisciplinary team member received an identifying code and was assured that the findings would be kept confidential.

## Results

### Preliminary analyzes

Univariate analyzes were conducted to select the appropriate control variables. Of these, only team’s tenure was negatively and significantly associated with team’s quality of care (see Table 1). In addition, *t*-test analysis revealed no significant differences in quality of care between for-profit and nonprofit institutions [t_(57)_ = −0.08; *p* > 0.05], and ANOVA analysis revealed that institute size was not significantly associated with team’s quality of care [*F*(2,55) = 2.07; *p* > 0.05]. However, in line with a previous study (Sheffer-Hilel et al., 2022), we decided to control for team’s tenure and team size, as these variables were associated with quality of care.

**Table 1:** Descriptive statistics and correlations between team’s quality of care and independent and control variables.

Characteristics		*M*	*SD*	1	2	3	4	5
1.	Team’s tenure	6.21	3.39	1				
2.	Professional stereotypes	0.56	0.25	−0.23^*^	1			
3.	Faultline	0.54	0.11	0.15^*^	−0.25^**^	1		
4.	Championship behavior	5.16	1.58	0.07	−0.60	−0.13^*^	1	
5.	Team’s quality of care	1.13	0.74	−0.02	0.11	−0.10	0.38^**^	1

*M*, mean; *SD*, Standard deviation. ^*^p < 0.05, ^**^p < 0.01.

### Hypotheses testing

Table 1 presents the correlations between the study variables. Championship behaviors were significantly associated with team’s quality of care (*r* = 0.38, *p* = 0.01). However, it was not significantly associated with professional stereotypes and faultlines. Table 2 shows the results of the linear mixed-model analysis for predicting team’s quality of care from the controls, independent variables, and their interactions. Step 1 included the controls (institute size and team’s tenure) and the independent variables of professional stereotypes, faultlines, and championship behaviors. Of these, only championship behaviors had a significant main effect on team’s quality of care (β = 0.22; *p* = 0.00).

**Table 2:** Results of the linear mixed-model analysis for predicting team’s quality of care from independent variables.

Characteristics	Step 1		Step 2		Step 3	
	*B*	(SE)	*B*	(SE)	*B*	(SE)
Institute size	0.01	0.11	0.05	0.11	0.07	0.10
Team’s tenure	0.00	0.02	0.01	0.03	0.00	0.02
Professional stereotypes	0.43	0.38	−0.59	2.58	16.96	10.75
Faultline	−0.18	0.81	9.33	3.89	27.38	11.40
Championship behavior	0.22^*^	0.07	1.00	0.44	2.97	1.25
Faultline × professional stereotypes			−0.189	3.56	−31.56	19.00
Faultline × championship behavior			−1.68*	0.62	−5.14	2.15
Professional stereotypes × championship behavior			0.28	0.34	−3.15	2.03
Faultline × professional stereotypes × championship behavior					6.09^*^	3.58
Δ-2Restricted Log Likelihood			13.65^a,**^		7.18^b,*^	
Institute-level variance	0.23	0.00	0.21	0.00	0.22	0.00
Residual	0.23^**^	0.09	0.21^*^	0.08	0.25^*^	0.01

*SE*, standard error. ^*^p < 0.05, ^**^p < 0.005.^a^The difference between Step 2 and Step 1 with 3 degrees of freedom. ^b^The difference between Step 3 and Step 2 with 1 degree of freedom.

**Figure 2:**
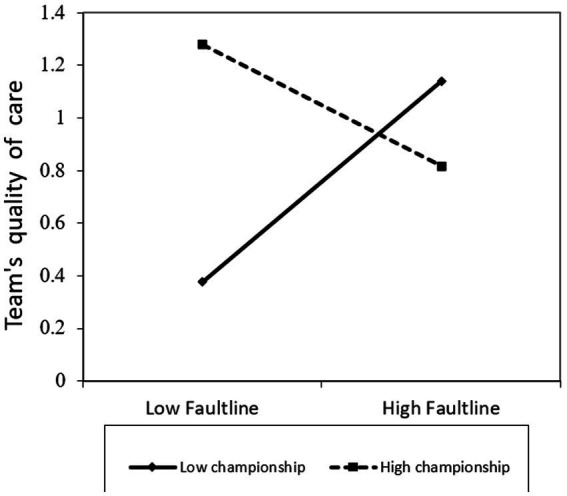
Team’s quality of care, by faultlines and championship behaviors.

In Step 2, only the two-way interaction effect of faultlines and championship behaviors on quality of care was significant (β = −1.68; *p* = 0.01). Figure 2 plots the two-way interaction effect on team’s quality of care. We followed the recommendations with values of 1 *SD* serving as weak faultlines and strong faultlines, respectively, (Dawson, 2014). As seen in Figure 2, when interprofessional teams have stronger faultlines, they will perform better when the leader engages in a low degree of championship behaviors, whereas when interprofessional teams have weak faultlines, they will perform better when the leader engages in a high degree of championship behaviors.

**Figure 3:**
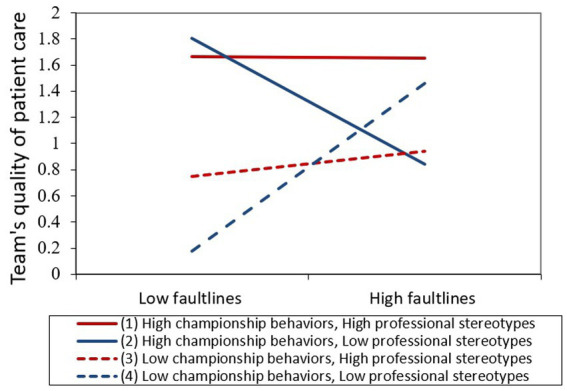
Team’s quality of care, by faultlines, and championship behaviors, and professional stereotypes.

The effect of the three-way interaction effect of faultlines, professional stereotypes, and championship behavior on team’s quality of care was significant (β = 6.09; *p* = 0.04). Figure 3 plots the three-way interaction on team’s quality of care. As demonstrated in the figure, when team’s professional stereotypes were high, team’s quality of care was positively associated with championship behaviors regardless of the level of faultlines. In comparison, when team’s professional stereotypes were low, team quality of care was contingent on championship behaviors such that when championship behaviors were low, the stronger the team faultlines, the higher the team’s quality of care, whereas when championship behaviors were high, the stronger the team faultlines, the lower the team’s quality of care, lending support to our hypotheses.

## Discussion

In healthcare, interprofessional teams represent an important strategy for boosting the quality of care, but in practice, teams often fail to benefit from their diversity (Homan et al., 2007; Mitchell and Boyle, 2019). Addressing this concern, we proposed that interprofessional teams will be less capable of gaining from their diversity when team stereotypes surface at the team level. By integrating leadership theory (Homan et al., 2020) and the CEM (van Knippenberg et al., 2004), we demonstrated that, in line with the CEM, when team stereotypes were high, social categorization processes were activated, and thus quality of care was critically dependent on the leader’s championship behaviors. When stereotypes were low, on the other hand, information elaboration processes were activated, and thus team faultlines were positively related to team’s quality of care. Moreover, under the latter condition, the leader’s championship behaviors could even harm the team’s quality of care. These novel findings contribute to the literature in several respects.

In line with recent recommendations in the field, we assessed team diversity in terms of team faultlines (Antino et al., 2019). The finding indicated that faultlines did not exert a significant direct effect on team quality of care. This finding supports recent developments in faultline theory, which argue that faultlines are not inherently “good” or “bad” (Bezrukova et al., 2010; Carton and Cummings, 2012; Meyer et al., 2015), and is in line with recent empirical findings that found no direct effect of faultlines on interprofessional team innovation (e.g., Mitchell and Boyle, 2020a).

This study also contributes to the interprofessional team literature by broadening the understanding of teams’ professional stereotypes. As we suggested, team members’ aggregated differences in the kinds of virtues attributed to colleagues based on their profession may represent this phenomenon as the property of the team. Our findings made it possible to examine professional stereotypes as a team-level phenomenon, thereby addressing recent calls to explore the impact of stereotypes on team performance (van Dijk et al., 2017). As Van Dijk et al. (2017) recommended, “More research that explicitly investigates the consequences of stereotypes in diverse teams is needed. To date, most research on the consequences of stereotyping has focused on the consequences for the target, and generally has not taken place in a team context” (p. 58). Conceptualizing team stereotypes at the team level may pave the way for further research on this issue.

Perhaps, our most intriguing finding is the significant three-way interaction effect of team faultline, team stereotypes, and championship behavior on team’s quality of care. As we found, team faultlines do not necessarily create team stereotypes (van Knippenberg et al., 2004). Further, when faultlines and team professional stereotypes were high, quality of care was not necessarily harmed. Instead, it was critically dependent on the leader’s championship behaviors. This finding supports the LeaD model (Homan et al., 2020), claiming that teams require different types of leadership in different circumstances. Apparently, when team stereotypes are high, leadership behaviors that are relationship-oriented are required for the team to run effectively. In this sense, championship behaviors that signal confidence in team success, encourage intergroup networking, and assign work to individual professionals without prejudice signal that all members of the team are regarded as equally important (Nembhard and Edmondson, 2006; Homan et al., 2020). Furthermore, the leader’s championship behaviors represent concomitant use of recategorization and decategorization strategies. Through recategorization, the leader creates an overarching, common, inclusive social identity through their demonstration of confidence in the group as a whole; through decategorization, the leader acknowledges and takes into account each individual’s contributions to the team by assigning the correct individual to the correct task without prejudice. In the end, these strategies enable leaders to sustain both their professional identity and their subordinate members’ team identity, thus overcoming the risk of threat to professional identity (Homan et al., 2020).

However, as our findings also revealed, when team faultlines are strong and professional stereotypes are low, the leader’s championship behavior can harm the relationship between faultlines and the team’s quality of care. Apparently, the team’s communication and coordination are unimpaired, so that the team may benefit from the elaboration of information stemming from the diverse perspectives each employee brings to the discussion. Under these circumstances, our findings showed, high championship behaviors are not only redundant but even harmful. This finding supports Homan’s LeaD and Kerr’s substitute-for-leadership theories, in that leadership style should fit a team’s needs (Kerr and Jermier, 1978; Homan et al., 2020). Preliminary empirical support for this argument was provided by studies on championship behaviors as well as the research on team’s professional diversity. As for the former, Walter et al. (2011) demonstrated that the leader’s championship behaviors could be too persistent in the face of adversity or take too much responsibility for an innovative undertaking, thus raising team members’ resistance to change. Similarly, Mitchell and Boyle’s (2020b) studies of interprofessional teams demonstrated that a relationship-oriented leadership style (e.g., inclusive leadership) had a negative effect on team outcomes when professional differentiation was low. Together, these findings highlight that a relationship-oriented leadership style is not productive for promoting quality of care for teams who do not face team bias or stereotypes.

### Limitations and recommendations for future research

This study has several limitations. First, as with similar research, there is merit in future research adopting longitudinal designs investigation of causal pathways. Second, despite our efforts to design a study embedded within a theoretical model, our study focused on person-related leadership (championship behaviors) and demonstrated that it fosters quality of care when stereotypes are high but hampers quality of care when stereotypes are low. Future research should explore whether task-related leadership can foster the interprofessional team’s quality of care when team stereotypes are low. Third, the nature of our healthcare sample LTCFs for elderly people was chosen precisely because of the importance of interprofessional teamwork in such facilities. Yet, the sample may be perceived as potentially limiting the generalizability of the findings. Although there is some evidence that healthcare teams face similar pressures across settings (Jeffcott and Mackenzie, 2008), future studies in different settings are warranted. Finally, we measured team quality of care as a process variable but made sure to use a different method–different source strategy, employing archival data. The study was conducted in LTCFs, where most residents had a complex medical condition and were in a poor cognitive state, and therefore unable to respond to questionnaires. Furthermore, the nutrition literature is equivocal in recommending clinical outcome measures to evaluate nutritional care in LTCFs (Moick et al., 2020). However, as improving quality of life is the primary objective of caring for the elderly, and as oral health and nutrition play a significant role in this, future research should consider developing measures of quality of care linked to older adults’ psychosocial outcomes (Rasheed and Woods, 2013; Porter et al., 2015; Joling et al., 2018).

### Practical implications

Our findings have important practical implications for managers and policymakers seeking to promote quality of care for patients *via* interprofessional teamwork. First, as our findings indicated, interprofessional team faultlines in themselves neither impeded nor improved a team’s quality of care, nor did team professional stereotypes. Our finding suggests that leaders should not try to create an inclusive team identity by any means, as previously recommended (Mitchell and Boyle, 2020a). Instead, they should help interprofessional team members develop a “dual team identity,” as members of both specific healthcare professions and interprofessional teams (Hill et al., 2019).

To this end, leaders should be well trained in analyzing team members’ needs: are team members currently struggling with stereotypes and prejudices hampering their performance? Or, alternatively, are they currently benefiting from the diverse opinions of each member? An assessment will subsequently identify the style of leadership that will foster the interprofessional team’s level of care. If the team tends to expend more energy on reinforcing existing stereotypes, the leader should assume a champion role by assigning the right specialists to assignments without bias, by signaling to coworkers that all are perceived as significant members of the group together, and by expressing confidence in the team’s success (Mitchell and Boyle, 2020b). Alternatively, if members of the interprofessional team communicate effectively without relying on stereotypes, another style of leadership may be required to ensure high-quality care.

It also logically follows that healthcare educators must already nurture dual identities in the early stages of students’ professional identity formation by providing both nonprofessional and interprofessional education throughout their programs. This may enable learners to understand their professional boundaries, and their contributions to an interprofessional team, without those boundaries developing into barriers, as they will not perceive their territories as being threatened. This may also ease the acceptance of those in new professions and discredit negative professional stereotypes (Hammick et al., 2007). Finally, these recommendations are of special importance for informal, low-status leaders of interprofessional teams, such as dietitians (Mak et al., 2019; Sheffer-Hilel et al., 2022). It is important to develop programs to teach those informal leaders how to become effective team leaders; such skills should be included in their academic training and continually reinforced during on-the-job training.

## Conclusion

Our findings make an important contribution to the understanding of team stereotypes as a property of the team and of the capacity of championship behaviors to mitigate the adverse impact of stereotypes on team’s quality of care. They highlight that team faultlines are not intrinsically harmful to team quality of care; instead, they can mitigate team quality of care only when team stereotypes emerge. Furthermore, the emergence of team stereotypes determines the type of leadership needed to promote quality of care. Whereas teams typified by high team stereotypes require a personal-relationship-oriented type of championship leadership, teams typified by low team stereotypes should apparently be treated with another type leadership style; otherwise, it can harm team’s quality of care. Taken together, the capacity of the team’s professional stereotypes to account for inconsistencies in the impact of interdisciplinary teams on quality of care, and the subsequent leadership style required, provide a direction for future research.

## Data availability statement

The raw data supporting the conclusions of this article will be made available by the authors, without undue reservation.

## Ethics statement

The studies involving human participants were reviewed and approved by the Ministry of Health’s Ethical Review Boards (24\2017) and the University’s Institutional Review Board approved the study (365/17). The patients/participants provided their written informed consent to participate in this study.

## Author contributions

GS, RE, and AD-Z developed the concept and study design. GS collected the data that was analyzed by GS, RE, and AD-Z. GS wrote the first draft with contributions from AD-Z. All authors contributed to the article and approved the submitted version.

## Conflict of interest

The authors declare that the research was conducted in the absence of any commercial or financial relationships that could be construed as a potential conflict of interest.

## Publisher’s note

All claims expressed in this article are solely those of the authors and do not necessarily represent those of their affiliated organizations, or those of the publisher, the editors and the reviewers. Any product that may be evaluated in this article, or claim that may be made by its manufacturer, is not guaranteed or endorsed by the publisher.
